# Breastfeeding restored the gut microbiota in caesarean section infants and lowered the infection risk in early life

**DOI:** 10.1186/s12887-020-02433-x

**Published:** 2020-11-25

**Authors:** Cheng Guo, Qian Zhou, Muxia Li, Letian Zhou, Lei Xu, Ying Zhang, Dongfang Li, Ye Wang, Wenkui Dai, Shuaicheng Li, Lin Zhang

**Affiliations:** 1 Department of Pediatrics, The Third Hospital of Hebei Medical University, No.139 Ziqiang Road, Shijiazhuang, 050051 China; 2 Department of Computer Science, City University of Hong Kong, Hong Kong, 999077 China; 3 School of Public Health, Peking University, No.38 Xueyuan Road, Beijing, 100191 China; 4 Department of Microbial Research, WeHealthGene Institute, Shenzhen, 518000 China; 5 Department of Information, The 960 Hospital of the Joint Logistic Support Force of the Chinese People’s Liberation Army, Jinan, 250031 China

**Keywords:** Gut microbiota, Early life, Meconium, Delivery mode, Feed pattern

## Abstract

**Background:**

The initialization of the neonatal gut microbiota (GM) is affected by diverse factors and is associated with infant development and health outcomes.

**Methods:**

In this study, we collected 207 faecal samples from 41 infants at 6 time points (1, 3, and 7 days and 1, 3, and 6 months after birth). The infants were assigned to four groups according to delivery mode (caesarean section (CS) or vaginal delivery (VD)) and feeding pattern (breastfeeding or formula milk).

**Results:**

The meconium bacterial diversity was slightly higher in CS than in VD. Three GM patterns were identified, including *Escherichia*/*Shigella*-*Streptococcus*-dominated, *Bifidobacterium*-*Escherichia*/*Shigella*-dominated and *Bifidobacterium*-dominated patterns, and they gradually changed over time. In CS infants, *Bifidobacterium* was less abundant, and the delay in GM establishment could be partially restored by breastfeeding. The frequency of respiratory tract infection and diarrhoea consequently decreased.

**Conclusion:**

This study fills some gaps in the understanding of the restoration of the GM in CS towards that in VD.

## Background

The intestinal tract hosts millions of microbial colonizers, and the gut microbiota (GM) is positively associated with human health [1, 2]. A wide variety of reports have demonstrated that caesarean section (CS) blocks gut and vaginal microbiota transmission from mothers to neonates, which delays subsequent health development [2, 3]. The feeding types also significantly shape the composition of the GM in infancy [4]. As human milk contains a high proportion of probiotics, prebiotics and active molecules, [5, 6] breastfeeding is more beneficial to GM maturation and health than formula feeding [7, 8].

Recent analyses have revealed that human milk promoted the functional maturation of GM after parturition [8]. A series of studies indicated that GM maturation was positively associated with pediatric health in the early life, named the window of opportunity [9–12]. Moreover, the delayed establishment of GM impacted infant development and increased the risks of disease pathogenesis during development [8, 13]. Considering the differences between Chinese and Western populations, such as differences in environment and diet, we collected 207 faecal samples from 41 Chinese neonates at six time points (in 24 h after birth, 48–72 h after delivery, and 7 days, 1, 3, and 6 months of age). We aimed to reveal whether breastfeeding could restore the GM established in CS towards that in vaginal delivery (VD) and lower the risk of infections in early life [7, 14].

## Methods

### Participant enrolment

The infants were enrolled from the Third Hospital of Hebei Medical University between Dec 2011 and Apr 2013. The inclusion criteria for mothers were as follows: i) no family allergy history; ii) no obesity, diabetes, allergic diseases, cardiovascular diseases or constipation during pregnancy; iii) full-term labour (> = 37 gestational weeks); iv) infants were fed by pure human milk (B group) or pure formula milk without prebiotics (F group). In combination with the mode of delivery (CS or VD), the enrolled children were assigned into four groups (VD_B, VD_F, CS_B and CS_F).

### Sample collection

During the regular examination at 6 time points (1, 3, and 7 days and 1, 3, and 6 months after birth), all faecal samples were collected under a nurse’s guidance using sample swabs (iClean, Huachenyang (Shenzhen) Technology Co., LTD, China) and stored in sterilized tubes (62–558-201, SARSTEDT AG & Co., KG, Germany). The collected samples were transferred to a − 80 °C freezer within 30 min after collection for long-term storage. Respiratory tract infection (RTI) and diarrhoea were recorded during the first year of life (Supplementary File 1). A total of 207 stool samples from 41 infants were collected between December 2011 and October 2014.

### DNA preparation and sequencing

Faecal bacterial DNA was extracted with the E.Z.N.A. DNA Kit (Omega BioTek, Norcross, GA, United States), and then, the V3–4 region of the 16S rDNA gene was amplified by the primers 338F (ACTCCTACGGGAGGCAGCAG) and 806R (GGACTACHVGGGTWTCTAAT) using a PCR kit (TransGenAP221–02, Peking, China). The verified amplicon products were then used to construct an amplicon library. Then, high-throughput DNA sequencing was conducted on the MiSeq platform (Illumina, San Diego, CA, United States).

### Bioinformatics analysis

The raw sequencing reads were filtered by Mothur software (v.1.43.0) with our in-house optimized scripts [15, 16]. The raw reads meeting any of the following criteria were removed: i) contained adapter sequences, ii) accumulated low-quality bases (lower than 20) at more than 10% of the read length. Then, the filtered paired reads were connected to tags with 10 bp overlaps. Tags were then clustered into operational taxonomic units (OTUs) using the Usearch method (v.10.0) [17]. Taxonomical annotation of OTUs was conducted using the RDP classifier (v.2.2) against the Greengenes database (v 13.5). Bacterial diversity was calculated by Mothur software, and the confounding effect of phenotypes was assessed through permutational multivariate analysis of variance (PERMANOVA). The stratification analysis of the delivery mode and feeding patterns was conducted by NMDS. The samples were assigned to the representative clusters based on the relative abundances of different microbial components according to the MetaHIT enterotype calculation method [18].

### Statistical analysis

The chi-square test was applied to analyse categorical variable differences, and one-way analysis of variance was used to assess continuous variables. The Wilcoxon rank-sum test was applied to evaluate significant differences in bacterial diversity and abundance between groups. Multiple statistical results from the Wilcoxon rank-sum test were adjusted with the Benjamini and Hochberg method (FDR < 0.05) using “p.adjust” in R (v. 3.6.0).

## Results

**Fig. 1:**
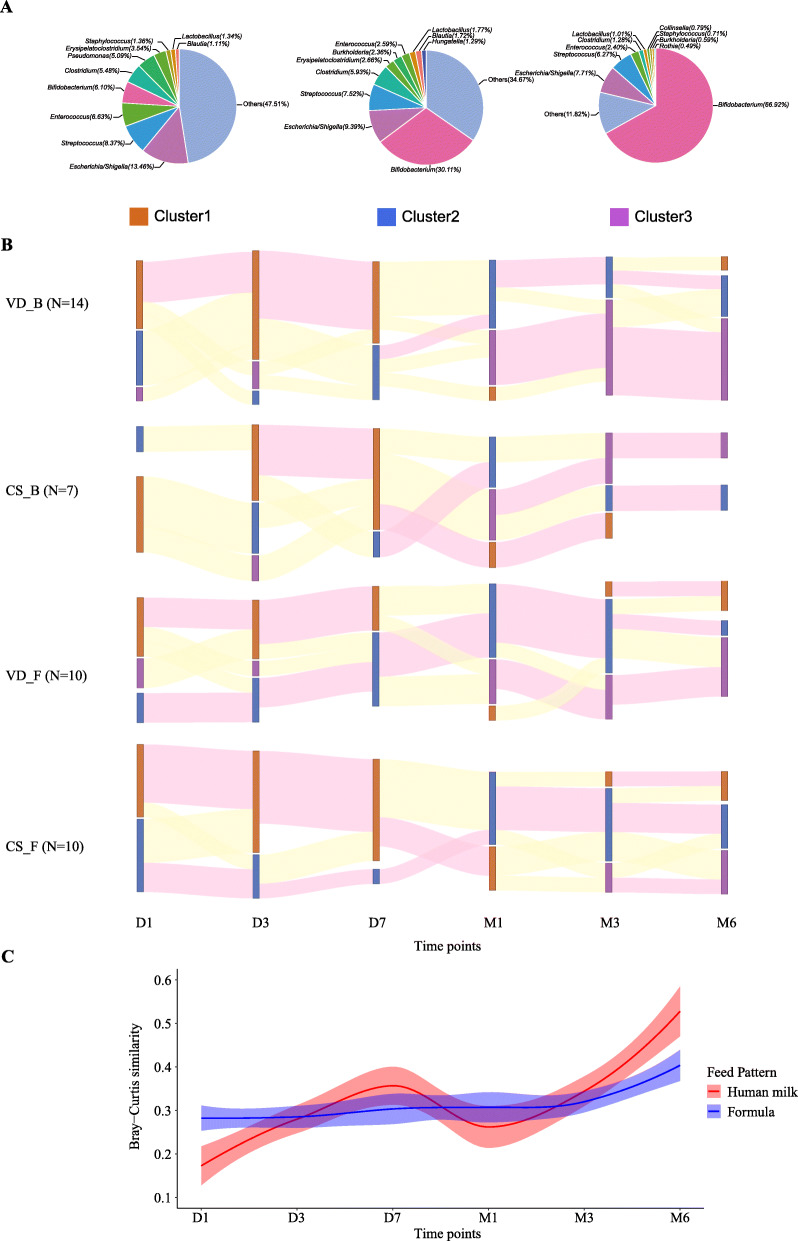
The patterns of the infant gut microbiota changed dynamically over time. **a** The GM clustered into 3 patterns. Each pie chart represents one GM pattern with the top 10 bacteria and others. GM Cluster 1 is *Escherichia/Shigella*-*Streptococcus* dominant and is coloured orange, Cluster 2 is *Bifidobacterium-Escherichia/Shigella* dominant and is coloured blue, and Cluster 3 is *Bifidobacterium* dominant and is coloured purple. **b** The dynamic change in GM patterns during the first half-year was different in four groups, including VD_B (vaginal delivery and breastfeeding), CS_B (caesarean section and breastfeeding), VD_F (vaginal delivery and formula feeding) and CS_F (caesarean section and formula feeding). The bar colour represents the GM pattern, and the bar length represents the proportion. The ribbon between bars indicates the changes in the GM pattern, where pink represents maintenance and yellow represents change. **c** Beta-diversity measured the difference in microbiota between CS and VD with age. Human milk (red colour) is better at restoring the infant gut microbiota than formula (blue colour)

All microbial samples were assigned to four groups according to delivery mode (VD and CS) and feeding pattern (breastfeeding, B; formula milk, F): VD_B (14 infants with 69 samples), VD_F (10 infants with 53 samples), CS_B (7 infants with 31 samples) and CS_F (10 infants with 54 samples) (Fig. 1, Table 1). There were no significant differences in infant gender, gestational age or mother’s age (Table 1, Supplementary File 1) between groups. Breastfeeding was significantly associated with a lower incidence of RTI and diarrhoea in both VD and CS infants (*P*-value < 0.001 and < 0.001, Table 1). In addition, PERMANOVA showed that the feeding pattern was the most dominant factor shaping the GM in the first 6 months (*P*-value =0.004).

**Table 1:** Characters’ distribution of 41 enrolled infants

	Breast feed	Formula feed	***p***-value
Delivery Mode			
Caesarean-section	7	10	0.279
Vaginal delivery	14	10	
Gender			
Female	7	12	0.087
Male	14	8	
Gestational age (week)^a^	39.18 ± 1.03	39.36 ± 1.19	0.615
RTI-frequency in the first year^a^	2.57 ± 1.03	4.05 ± 1.06	< 0.001
Diarrhea-frequency in the first year^a^	1.14 ± 0.79	2.35 ± 1.09	< 0.001

^a^Represented by mean ± SD

Although insignificant, the GM diversity in CS neonates (3.18 ± 0.68) was higher than that in VD neonates (3.01 ± 1.51) at six time points (Supplementary File 2A). Compared to CS infants, *Bifidobacterium* was enriched nearly two-fold in the VD infants’ meconium (20.70% ± 20.01) (Supplementary File 3). Other accumulated microbial components in CS infants included *Escherichia/Shigella*, *Enterococcus*, *Streptococcus*, *Burkholderia*, *Acinetobacter*, *Lactobacillus* and *Ralstonia* (Supplementary File 3).

The 207 faecal samples collected were classified into 3 clusters according to GM structure. *Escherichia/Shigella* and unclassified taxa dominated the GM in Cluster 1, while *Bifidobacterium* and unclassified taxa were dominant in the GM of Cluster 2 (Fig. 1a). In Cluster 3, *Bifidobacterium* was the most abundant genus in the GM (Fig. 1a). In the first week, the Cluster1 GM pattern was identified in most of the samples (Fig. 1b), and the relative abundances of *Enterococcus* and *Escherichia/Shigella* increased slightly (Supplementary File 3). The abundance of the meconium-dominant *Pseudomonas* decreased sharply on day 3, especially in infants receiving breastfeeding (*P-value* = 0.004, 0.028 in VD_B and CS_B) (Supplementary File 3). During the neonatal period, especially from D7 to M1, the GM composition shifted from Cluster 1 to Cluster 2 or Cluster 3 (Fig. 1b). *Bifidobacterium* was significantly enriched in the GM of breastfeeding infants (*P-value* = 0.004, 0.028 in VD_B and CS_B) (Supplementary File 3). The CS_F group contained the most abundant unclassified taxon and the lowest *Bifidobacterium* load in the GM, while VD_B infants had the opposite trend. When receiving breastfeeding, the GM similarity between CS and VD infants was higher (from 0.18 to 0.52) than that with infants who experienced formula feeding (Fig. 1c).

## Discussion

The assemblage of the GM during infancy is derived from the mother’s faecal, vaginal and skin microbiota [19]. GM structures change dynamically over time in early life [20–22]. Facultative anaerobic bacteria, such as *Escherichia* and *Streptococcus*, colonize the infant intestinal tract, consuming oxygen in the first few days after delivery, and then strict anaerobes, especially *Bifidobacterium,* thrive in the GM [3]. In this study, we identified 3 GM profiles that were dominated by an unclassified *Escherichia/Shigella* taxon, an unclassified *Bifidobacterium* taxon or *Bifidobacterium*. The GM pattern gradually changed from Class 1 to Class 3, which is consistent with prior reports [21].

Maternal milk contains abundant nutrients, such as prebiotics, as well as beneficial bacteria, such as *Bifidobacterium* [6, 23]. The key role of human milk in GM maturation has been previously emphasized [6, 7]. The enriched *Bifidobacterium* sp. could degrade human milk oligosaccharides (HMOs) [5, 24] to produce lactate and acetate, which maintain a low pH for digestive enzyme activation and serve as energy sources for colonocytes [25]. Human milk also facilitates later colonization of anaerobic microbial commensals, educating the host immune system and providing colonization resistance for opportunistic pathogens [24].

The positive contribution of human milk to GM development [5, 6] may partly explain why breastfeeding could restore the delayed GM development in CS infants towards that in VD as well as lower the risk of RTI and diarrhoea [26] Consistent with prior findings that GM development is successive, [20, 27] our study also identified no specific time point for breastfeeding-associated GM restoration.

Despite the additional insight into the GM restoration caused by breastfeeding, several limitations of our study should be noted. A small sample size may cause some bias in the analysis, and we are conducting a multicentre longitudinal study to confirm our preliminary findings. In an on-going project, we also enrolled infants who were fed formula with probiotics to confirm whether additive probiotics could better improve GM maturation and lower the risk of diseases.

This study revealed that breastfeeding could restore the delayed GM development of caesarean infants. The results expand the understanding of dynamic changes in the GM that occur in early life and provide new evidence to support the breastfeeding policy.

## Supplementary Information

**Additional file 1.**

**Additional file 2.**

**Additional file 3.**

## Data Availability

The datasets generated and analysed during the current study are available in the GenBank database under accession number PRJNA576564, http://www.ncbi.nlm.nih.gov/genbank/.

## Abbreviations

CS: Caesarean-section
GM: Gut microbiota
OTUs: Operational taxonomic units
PERMANOVA: Permutational multivariate analysis of variance
RTI: Respiratory tract infection
VD: Vaginal delivery
