# Supplementary figures and images for: Breastfeeding restored the gut microbiota in caesarean section infants and lowered the infection risk in early life
Source: BMC Pediatr. 2020 Nov 25;20:532. doi: 10.1186/s12887-020-02433-x (PMC7690020; doi:10.1186/s12887-020-02433-x)

A

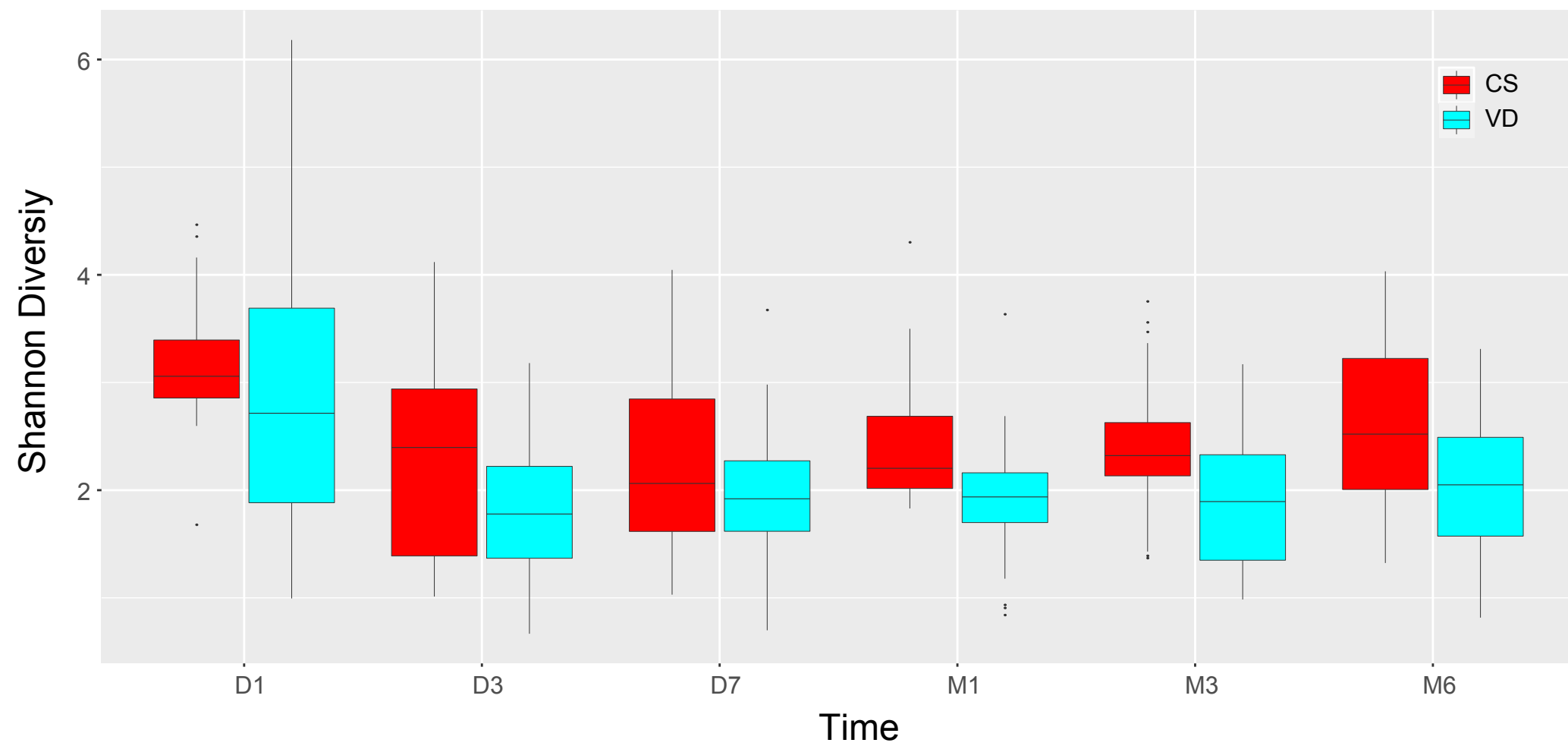

B

Feed Pattern

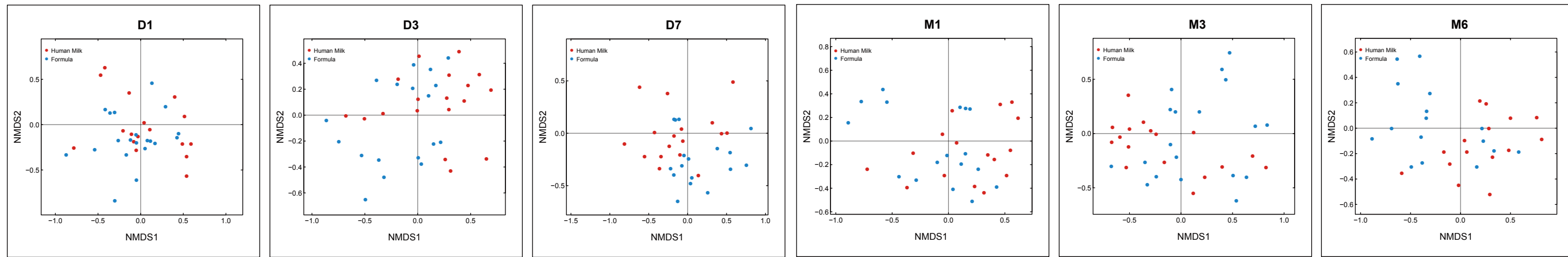

Delivery Mode

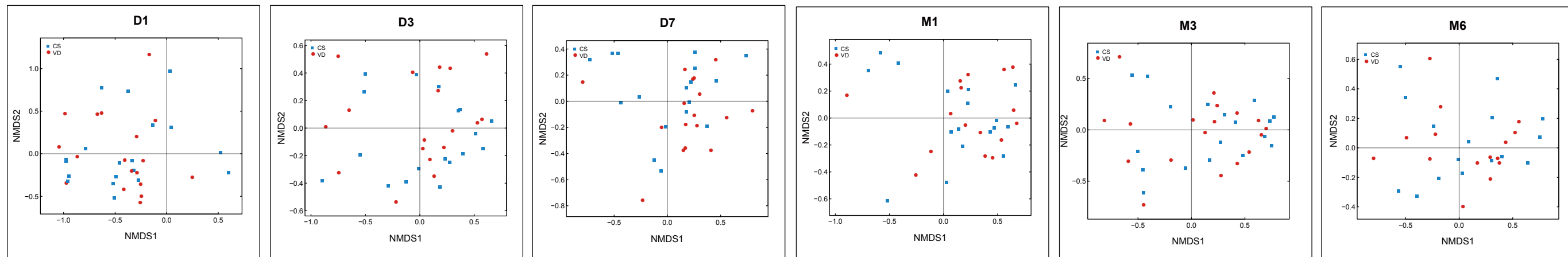

Supplement: Supplementary file 2 — Additional file 2. [file 12887_2020_2433_MOESM2_ESM.pdf]
